# A Facile Electrochemical Preparation of Reduced Graphene Oxide@Polydopamine Composite: A Novel Electrochemical Sensing Platform for Amperometric Detection of Chlorpromazine

**DOI:** 10.1038/srep33599

**Published:** 2016-09-21

**Authors:** Selvakumar Palanisamy, Balamurugan Thirumalraj, Shen-Ming Chen, Yi-Ting Wang, Vijayalakshmi Velusamy, Sayee Kannan Ramaraj

**Affiliations:** 1 Electroanalysis and Bioelectrochemistry Lab, Department of Chemical Engineering and Biotechnology, National Taipei University of Technology, No. 1, Section 3, Chung-Hsiao East Road, Taipei 106, Taiwan ROC; 2 Division of Electrical and Electronic Engineering, School of Engineering, Manchester Metropolitan University, Manchester M1 5GD, United Kingdom; 3 PG & Research department of Chemistry, Thiagarajar College, Madurai, 625009, India

## Abstract

We report a novel and sensitive amperometric sensor for chlorpromazine (CPZ) based on reduced graphene oxide (RGO) and polydopamine (PDA) composite modified glassy carbon electrode. The RGO@PDA composite was prepared by electrochemical reduction of graphene oxide (GO) with PDA. The RGO@PDA composite modified electrode shows an excellent electro-oxidation behavior to CPZ when compared with other modified electrodes such as GO, RGO and GO@PDA. Amperometric *i-t* method was used for the determination of CPZ. Amperometry result shows that the RGO@PDA composite detects CPZ in a linear range from 0.03 to 967.6 μM. The sensor exhibits a low detection limit of 0.0018 μM with the analytical sensitivity of 3.63 ± 0.3 μAμM^–1 ^cm^–2^. The RGO@PDA composite shows its high selectivity towards CPZ in the presence of potentially interfering drugs such as metronidazole, phenobarbital, chlorpheniramine maleate, pyridoxine and riboflavin. In addition, the fabricated RGO@PDA modified electrode showed an appropriate recovery towards CPZ in the pharmaceutical tablets.

Graphene has continuously received significant scientific interest from the theoretical chemists since the pioneering work of Geim and Novoselov *et al.* in 2004^1^. As an alternative material to pristine graphene, a two dimensional (2D) carbon nanomaterial reduced graphene oxide (RGO) is also widely used for different applications owing to its distinct properties such as high surface area combined with remarkable mechanical, thermal and electrical properties^2^,^3^,^4^,^5^,^6^,^7^. To date, different reduction protocols have been employed for efficient reduction of graphene oxide (GO) into RGO such as, thermal reduction^8^, microwave and photo^9^, photo catalyst^10^, chemical^11^, solvothermal^12^ and electrochemical reduction methods^13^. Compared with the aforementioned reduction protocols, electrochemical reduction methods are found relatively simple, cost-effective, fast and environmentally friendly^14^. In addition, it is reported that electrochemically prepared RGO has retains some of the original properties of pristine graphene with new functionalities of RGO^15^.

Polydopamine (PDA) is a significant pigment of naturally occurring melanin (eumelanin) and has interesting of optical, electrical, and magnetic properties combined with excellent biocompatibility^16^,^17^,^18^. PDA also serves as a functional material for incorporating the various functional groups such as catechol, amine, and imine^19^,^20^. PDA can be easily incorporated within GO or RGO as a functional nanomaterial in wide range of biomolecular and electrochemical sensor applications^21^,^22^,^23^. Chlorpromazine (CPZ) is well-known antipsychotic drug, widely used in the treatment of schizophrenia, bipolar disorder and mania^24^,^25^. It is reported that the possible of risk of cancer is higher in patients undergoing continuous treatment with CPZ^24^. Therefore, the accurate and reliable detection of CPZ is essential to determine exposure levels.

To date, only a limited number of papers have focused on RGO@PDA composite for electrochemical sensor applications and all reported techniques for the fabrication of PDA incorporated RGO have done so by chemical methods^22^,^23^. In the present study, we have fabricated an RGO@PDA composite by a simple electrochemical method for the first time and utilized it as an advanced electrocatalyst for the oxidation of CPZ.

Herein, we report a selective and sensitive amperometric detection of CPZ using a novel composite of RGO@PDA modified electrode. The RGO@PDA composite shows a greater catalytic activity toward CPZ when compared with other modified electrodes. The stability, selectivity, and reproducibility of the sensor has been characterized and discussed in detail. The viability of the sensor has also been verified and critically assessed with pharmaceutical tablets containing the active ingredient, CPZ. The schematic representation for electrochemical fabrication of RGO@PDA composite is shown in Fig. 1.

## Results and Discussion

### Characterizations

Figure 2 displays the SEM images of GO (A), GO@PDA (B), RGO (C) and RGO@PDA composite (D). The SEM image of GO shows its typical crumbled morphology with the associations of several nano graphite layers. The GO@PDA composite highlights an individual GO sheets with a thin layer of PDA embedded on the surface of GO without aggregation. The SEM image of RGO shows a creased and disintegrated morphology with ultra-thin RGO nanosheets that are randomly aggregated. In contrast, RGO@PDA composite shows a 3D morphology, where a thin layer of PDA uniformly covered the surface of RGO sheets. This may be attributable to the strong hydrophilic nature of DA entrapped directly on to the RGO sheets.

The presence of RGO and PDA was further confirmed by elemental analysis, and the corresponding elemental mapping is shown in Fig. 3B–D. The elemental mapping results confirmed the presence of carbon (B), oxygen (C) and nitrogen (D) in RGO@PDA composite, indicating the presence of RGO and PDA.

The FT-IR and Raman spectroscopy confirms formation of the RGO@PDA composite. Raman spectroscopy is considered an ideal and informative technique for the characterization of carbon materials^26^. The G band in Raman spectra is resulting to the doubly degenerate phonon mode at the Brillouin zone center of sp^2^ carbon networks, while the D band is due to the disorder-induced phonon mode corresponding to scattering on the K-point phonon of sp^2^ carbon networks^27^. Figure 4A shows the typical Raman spectrum of GO (a), GO@PDA (b), RGO (c) and RGO@PDA composite (d). GO shows the D and G band at 1356 and 1593 cm^−1^, while, D and G band of GO@PDA is observed at 1358 and 1593 cm^−1^. The RGO@PDA composite shows D and G band at 1339 and 1604 cm^−1^, and the observed D and G bands are more consistent with Raman spectra of RGO. The observed shift of D and G bands in RGO@PDA composite is likely to result from the reduction of GO. The intensity ratio of D to G bands (I_D_/I_G_) of GO, GO@PDA, RGO and RGO@PDA were calculated as 0.92, 0.9, 1.04 and 1.02, respectively. The I_D_/I_G_ results indicate the transformation of GO/PDA to RGO/PDA, and the I_D_/I_G_ of RGO is consistent with those previously reported in the literature^27^.

FT-IR spectroscopy further confirms the formation of RGO@PDA composite. Figure 4B shows the FT-IR spectra of GO (a), GO@PDA (b) and RGO@PDA (c) composite. It can be seen that GO exhibits the bands at 3395, 2906, 1638, 1412, 1214 and 1051 cm^−1^ is corresponding to the vibrations of O-H stretching; vibration of C-H; bending vibration of adsorbed water; deformation of C–O and vibration modes of epoxide (C-O-C) respectively^28^. The result confirms the successful oxidation of graphite to GO. FT-IR of GO@PDA also shows a new characteristic peak at 1378 cm^–1^, ascribed to the vibration of phenolic C-O-H bending in PDA^29^. Furthermore, two distinguishing vibration peaks were observed at 1468 and 1378 cm^–1^, associated with the stretching vibration of C=N and C-N-C from PDA^30^. The bands at 1730, 1378 and 3811 cm^–1^ are due to the phenolic C-OH stretching vibration and O-H stretching vibrations of PDA^30^. The result confirms the formation of GO@PDA composite. In contrast, RGO@PDA composite exhibits the bands at 1292 and 1446 cm^–1^, associated with the vibration of phenolic C-O-H and stretching vibration of C=N from PDA^30^. Those bands related to -OH vibrations were significantly removed in RGO@PDA composite, which indicates efficient electrochemical reduction of GO@PDA to RGO@PDA.

### Electrochemical behavior of CPZ

The electrochemical behavior of CPZ at different modified electrodes was investigated by CV. Figure 5 depicts the CV response obtained for GO (a), GO@PDA (b), RGO (c) and RGO@PDA (d) modified electrodes in the presence of 2 mM CPZ in N_2_ saturated PBS at a scan rate of 50 mV/s. The GO modified electrodes show a weak oxidation peak current response for CPZ at 0.71 V. The GO@PDA modified electrode shows an enhanced oxidation peak current response for CPZ at 0.735 V when compared to GO modified electrode. The result indicates that GO@PDA has enhanced detection of CPZ over the GO modified electrode. In contrast, the oxidation peak of CPZ was observed at 0.72 V in RGO modified electrode, and the current response was smaller than those observed in GO@PDA, which indicates that PDA has played significant role towards the enhancement of oxidation peak of CPZ. However, the RGO@PDA composite shows an oxidation peak at 0.763 V for CPZ. The oxidation peak current response of CPZ at RGO@PDA composite was 8.5, 2.5 and 4.4 fold higher than those detected at GO, GO@PDA and RGO modified electrodes (Fig. 5 inset) respectively. The enhanced activity of RGO@PDA composite towards CPZ may be due to the enrichment effect of PDA and high surface area of RGO. PDA serves as a possible matrix for adsorption of more CPZ on the electrode surface via strong π-π interaction between PDA and CPZ. The electron transfer between CPZ and electrode surface was further increased by the presence of RGO under diffusion conditions, thus resulting in enhanced oxidation peak current response to CPZ at the RGO@PDA composite modified electrode. The results indicate that RGO@PDA composite is more effective in the detection of CPZ when compared to other modified electrodes.

CV was applied to investigate the effect of scan rate on the electrochemical performance of CPZ at RGO@PDA composite modified electrode. Figure S1 shows the CV response of RGO@PDA composite modified electrode in 2 mM CPZ containing N_2_ saturated PBS at different scan rate from 20 to 200 mV/s (a–i). The oxidation peak current response of CPZ is directly proportional to the scan rate. Additionally, when the scan rates were increased from 20 to 200 mV/s, the peak potential underwent a negative shift. The anodic peak current of CPZ showed a linear relationship with the scan rate square root from 20 to 200 mV/s with a correlation coefficient of 0.9983 (Fig. S1 inset). It is evident from the results that the electrochemical oxidation of CPZ at RGO@PDA composite modified electrode is a diffusion controlled process^31^. Since the pH is crucial factor that can dramatically change the electrocatalytic activity of the modified electrode, the effect of pH on the oxidation peak current of CPZ was investigated by CV for the RGO@PDA composite modified electrode. Figure S2 shows the CV response obtained at RGO@PDA composite modified electrode in N_2_ saturated PBS containing 2 mM CPZ, at pH 3, pH 5, pH 7 and pH 9 containing 2 mM CPZ at a scan rate of 50 mV/s. A maximum oxidation peak current response of CPZ was observed at pH 7 (Fig. S2 inset) hence, pH 7 was used as an optimum pH for further experiments.

The electrocatalytic activity of the RGO@PDA composite modified electrode towards the oxidation of CPZ was studied by CV. Figure S3 displays the cyclic voltammetric response of RGO@PDA composite modified electrode in the absence (a’) and presence of concentrations of CPZ from 0.3–2.0 mM in N_2_ saturated PBS at a scan rate of 50 mV/s. In the absence of CPZ, no response was observed at the RGO@PDA composite electrode during the potential scanning from 0.1 to 1.0 V, indicating the composite modified electrode is electrochemically inactive in this potential range. In the presence of 0.3 mM (a) of CPZ, a sharp oxidation peak response was observed at 0.731 V. The oxidation peak current response of CPZ increases with the further addition of CPZ ((b) 0.7, (c) 1.0, (d) 1.5 and (e) 2.0) into the PBS, while the oxidation peak shifts towards positive direction. The electrochemical oxidation mechanism of CPZ at carbon modified electrodes is well studied and reported^32^. According to the early reports, it is believed that the electrochemical oxidation of CPZ occurs at nitrogen atoms, which corresponds to the oxidation peak of CV at 0.731 V. The electrochemical oxidation mechanism of CPZ at RGO@PDA composite modified electrode is shown in Fig. 6.

### Amperometric determination of CPZ

To determine CPZ using RGO@PDA composite modified RDE, an amperometric *i-t* method was employed under optimum conditions. Figure 7 shows the amperometric *i-t* response for RGO@PDA composite modified RDE at different concentrations (0.003 to 1117.6 μM) of CPZ in constantly stirred N_2_ saturated PBS at a working potential of 0.8 V. A stable and well-defined amperometric *i-t* response was observed for the addition of 0.03 (a), 0.3 (b), 1 (c), 5 (d), 10 (e), 50 (f) and 100 μM (g) of CPZ into the PBS (Fig. 7 upper inset). Notably, the RGO@PDA composite modified electrode shows a sharp amperometric response for the addition of 0.03 (a) and 0.3 μM (b) of CPZ. The response time of the CPZ sensor was determined as 4 s, indicating the fast diffusion of CPZ on the modified electrode surface. The RGO@PDA composite modified electrode shows a stable amperometric response in a linear concentration range from 0.03 to 967.6 μM (Fig. 7 lower inset). The analytical sensitivity (slope/electrode active surface area) of the CPZ sensor was calculated as 3.63 ± 0.3 μAμM^–1 ^cm^–2^ with a detection limit (LOD) of 0.0018 μM based on S/N = 3 (3∗standard deviation of the blank response)/slope of the calibration plot, Sd = 0.001 and slope = 0.167). The RGO@PDA composite modified electrode shows a lower LOD (1.8 nM) and a wider linear response (up to 967.6 μM) to CPZ when compared to the LOD and linear response range of graphene paste electrode (3 nM and 9.0 μM)^32^, carbon nanotube-polyethyleneimine electrode (10 nM and 115.4 μM)^33^, poly-3-methylthiophene combined with γ-cyclodextrin composite electrode (0.2 μM and 3 μM)^34^ and Alizarin Red modified electrode (5.16 μM and 500.0 μM)^25^. In addition, the observed sensitivity (3.63 ± 0.3 μAμM^–1 ^cm^–2^) of the present sensor is higher than all aforementioned modified electrodes for the detection of CPZ. However, the LOD of the present sensor is higher than the LOD (0.01 nM) of previously reported GO based electrochemiluminescence CPZ sensor^35^. The result confirms that the RGO@PDA composite modified electrode may be used for the sensitive and low level electrochemical detection of CPZ.

### Selectivity of the sensor

Evaluation of sensor selectivity is more important in real samples since other drugs can potentially interfere with the amperometric signal of CPZ. Therefore, amperometric *i-t* method was used to evaluate the selectivity of the RGO@PDA composite modified electrode. Figure S4A shows the amperometric *i-t* response obtained at RGO@PDA composite modified RDE for the addition of 1 μM of CPZ (a) and 100 μM of metronidazole (b), phenobarbital (c), chlorpheniramine maleate (d), pyridoxine (e), riboflavin (f) and ascorbic acid (g) into constantly stirred N_2_ saturated PBS at a working potential of 0.8 V. A well-defined amperometric response was observed for each addition of 1 μM CPZ, while 100 μM additions of potentially interfering drugs did not show any amperometric response on RGO@PDA composite modified electrode. The result indicates the high selectivity of RGO@PDA composite modified electrode towards CPZ, which is due to the strong π-π interaction between PDA and CPZ than that of other drugs. The high selectivity of RGO@PDA composite modified electrode further confirmed its use in the selective detection of CPZ in real samples.

### Determination CPZ in pharmaceutical tablets

In order to evaluate the practicability of RGO@PDA composite towards CPZ, the modified electrode was evaluated for detection of CPZ in pharmaceutical tablets using amperometry. The experimental conditions are similar as of in Fig. 7. Prior to real sample analysis, the WINSUMIN (12.5 mg) CPZ tablet stock solutions were prepared by dissolving an appropriate number of WINSUMIN tablets into PBS. A known concentration of real sample was spiked into PBS and used for real sample analysis. Prior to analysis, an un-known concentration of CPZ in WINSUMIN tablets is determined as 28.6 μM by using standard addition method^32^ and the same method was used to determine the detection and the recovery of CPZ as listed in Table ST1. The RGO@PDA composite modified electrode shows a recovery of CPZ ranging from 98.5 to 99.2% in WINSUMIN tablets. The result validates that RGO@PDA composite modified electrode has a satisfactory recovery towards CPZ and may be used for the quantification of CPZ in pharmaceutical tablets.

### Stability, precision and accuracy of the sensor

The operational stability of CPZ sensor was evaluated by amperometry. Figure S4B shows amperometric *i-t* response of RGO@PDA composite modified RDE for addition of 50 μM of CPZ (a) into constantly stirred N_2_ saturated PBS and its background current response up to 2000 s. The RGO@PDA composite modified electrode retains 93.2% of initial response of CPZ even after continuous run up to 2000 s and the result indicates good operational stability of the developed CPZ sensor. The storage stability was evaluated by CV under similar experimental conditions as in Fig. 5. The RGO@PDA composite modified electrode lost only 7.2% of initial current response to CPZ following storage in 2 mM containing PBS, indicating the satisfactory storage stability of the modified electrode. The reproducibility and repeatability of RGO@PDA composite modified electrode were investigated using CV and working conditions are similar as of in Fig. 5. The relative standard deviation (RSD) of 3.9% was found for three RGO@PDA composite modified electrodes for detection of 2 mM CPZ. An RSD of approximately 4.6% was observed for 10 measurements of a single RGO@PDA composite modified electrode in 2 mM CPZ containing PBS. These results indicate that RGO@PDA composite modified electrode has an appropriate reproducibility and repeatability for the detection of CPZ.

## Conclusions

In conclusion, a novel RGO@PDA composite has been prepared by a simple electrochemical method and used for selective amperometric detection of CPZ. SEM, Raman and FTIR results confirm formation of the RGO@PDA composite. The RGO@PDA composite modified electrode showed greater catalytic activity towards CPZ than other modified electrodes. It showed excellent analytical characteristics for the detection of CPZ lower LOD (0.0018 μM), fast response (4 s), high sensitivity (3.63 ± 0.3 μAμM^–1 ^cm^–2^) and wider response range (up to 967.6 μM). In addition, RGO@PDA modified electrode had a high selectivity towards CPZ in the presence of potentially interfering other drugs. Effective recovery of CPZ in pharmaceutical tablets further confirms RGO@PDA composite modified electrode as a suitable electrode material for the detection of CPZ. In addition, we believe that the novel RGO@PDA composite represents an attractive material for applications in biomedical and sensor fields.

## Experimental

### Materials and Methods

Chlorpromazine hydrochloride (≥98%) was obtained from Sigma–Aldrich and used as received. Dopamine was purchased from Sigma–Aldrich. Graphite power with an average diameter of about >20 μm was obtained from Sigma–Aldrich. NaH_2_PO_4_ was purchased from Sigma–Aldrich Company and Na_2_HPO_4_ was obtained from Avantor Performance Materials Inc, Center Valley, U.S.A. The WINSUMIN 12.5 mg chlorpromazine tablets were purchased from a local pharmaceutical company in Taipei, Taiwan. The WINSUMIN tablet samples were prepared by dissolving an appropriate amount of WINSUMIN tablets into phosphate buffer solution (PBS) pH 7.0. All other chemical were used in this study were obtained from Aldrich and the solutions were prepared using doubly distilled water without any further purification. The supporting electrolyte 0.05 mol L^–1 ^pH 7 PBS solution was prepared by using 0.05 mol L^–1^ Na_2_HPO_4_ and NaH_2_PO_4_ solutions in double distilled water.

Cyclic voltammetry (CV) and amperometric *i-t* measurements were performed by computerized CHI 1205b electrochemical work station. Scanning electron microscopic (SEM) images were taken using Hitachi S-3000 H electron microscope. Raman spectra were acquired using a Raman spectrometer (Dong Woo 500i, Korea) equipped with a charge-coupled detector. Fourier transform infrared spectroscopy (FT-IR) was carried out using the Thermo SCIENTIFIC Nicolet iS10 instrument. HORIBA EMAX X-ACT attached Hitachi S-3000 H scanning electron microscope was used to perform energy-dispersive X-ray spectrum and elemental mapping. Amperometric *i–t* measurements were performed with an analytical rotator AFMSRX (PINE instruments, USA). Typical three electrode setup was used for electrochemical measurements, where glassy carbon electrode as a working electrode, a saturated Ag/AgCl reference electrode and a platinum electrode as an auxiliary electrode. A rotating disk electrode (RDE) with an electrochemically active surface area (EASA) of 0.046 cm^2^ was used for amperometric *i-t* measurements. The EASA of RGO@PDA composite modified RDE was calculated by CV, as reported early^36^. All electrochemical measurements were carried out at a room temperature in an inert atmosphere unless otherwise stated.

### Synthesis of RGO@PDA composite

Graphite oxide was prepared from graphite powder by Hummers method as reported elsewhere^28^. The GO@PDA composite was prepared by our previously reported method^37^. Briefly, graphene oxide (GO) solution was prepared by dispersing of graphite oxide (2 mg mL^–1^) into DI water and followed by sonication for 30 min. Approximately 10 mg of DA was added into GO solution (10 mL) and solution pH was adjusted to 8.5 using 40% NH_4_OH under magnetic string. The reaction mixture was continuously stirred up to 2 h in room temperature. According to early reports, DA can be self-polymerized to form a PDA thin film directly on surface of GO^37^. The obtained GO@PDA composite was further purified and separated by filtration. The final GO@PDA composite was washed with DI water and ethanol, dried in an air oven, and re-dispersed in DI water for experimental use.

**Figure 1:**
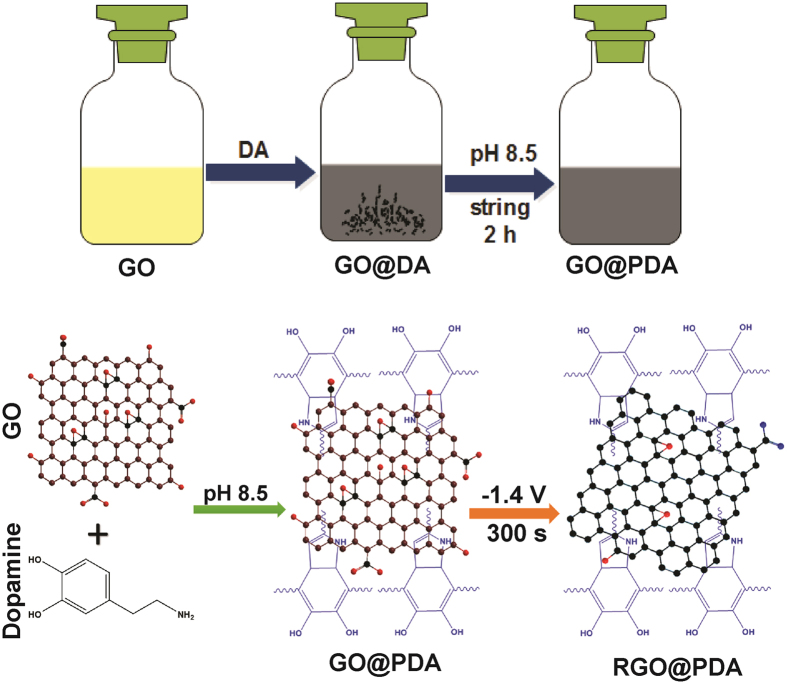
Schematic representation for the electrochemical fabrication of RGO@PDA composite.

**Figure 2:**
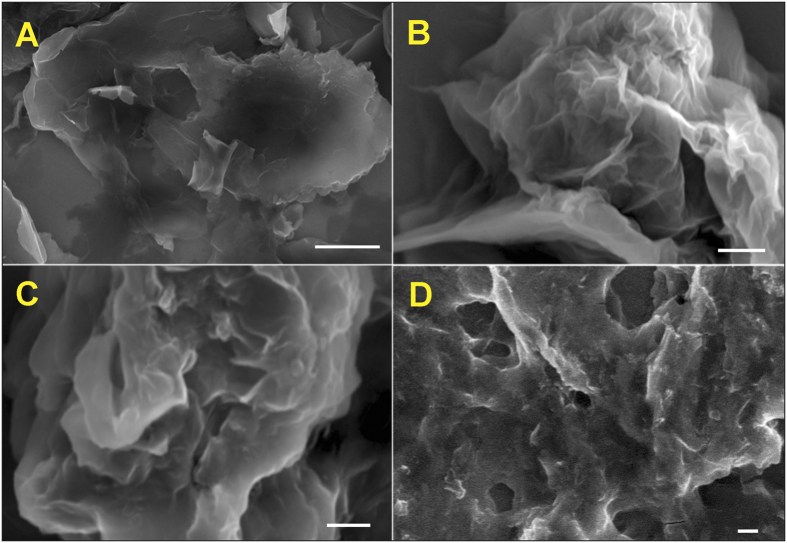
SEM images of GO (**A**), GO@PDA (**B**), RGO (**C**) and RGO@PDA composite (**D**). Scale bar = 2 μM.

**Figure 3:**
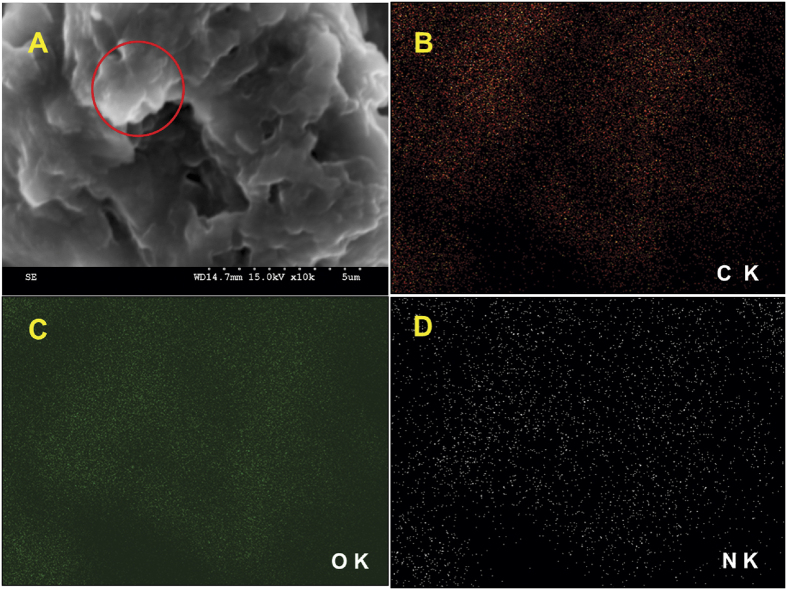
(**A**) SEM image of RGO@PDA composite and corresponding elemental mapping of carbon (**B**), oxygen (**C**) and nitrogen (**D**).

**Figure 4:**
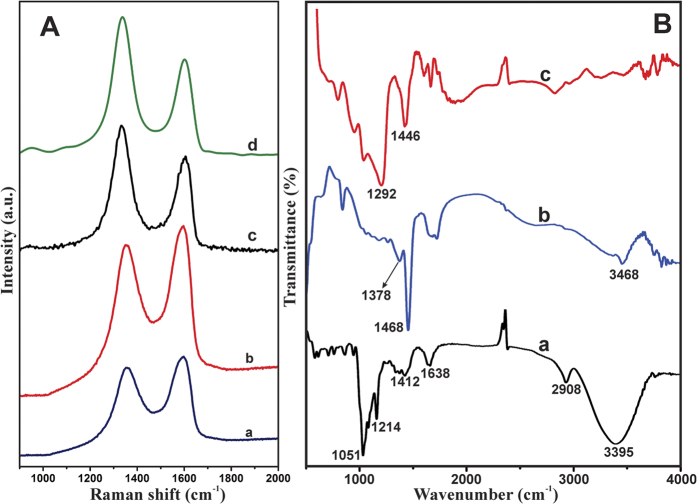
(**A**) Raman spectra of GO (a), GO@PDA (b), RGO (c) and RGO@PDA composite (d). (**B**) FT-IR spectra of GO (a), GO@PDA (b) and RGO@PDA composite (c).

**Figure 5:**
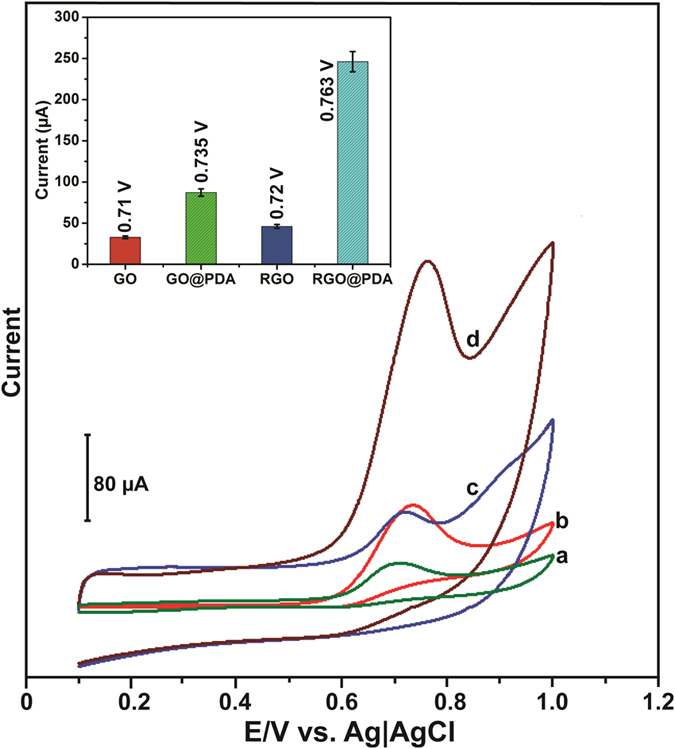
CV response of GO (a), GO@PDA (b), RGO (c) and RGO@PDA composite (d) modified electrodes in the presence of 2 mM CPZ in N_2_ saturated PBS at a scan rate of 50 mV/s. The potential of CPZ and the oxidation peak current response at different modified electrodes are shown in the Inset. The error bars are relative to five measurements.

**Figure 6:**
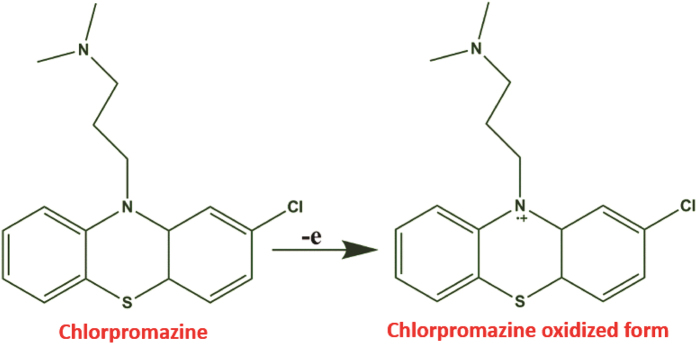
Electro-oxidation mechanism of CPZ at RGO@PDA composite modified electrode.

**Figure 7:**
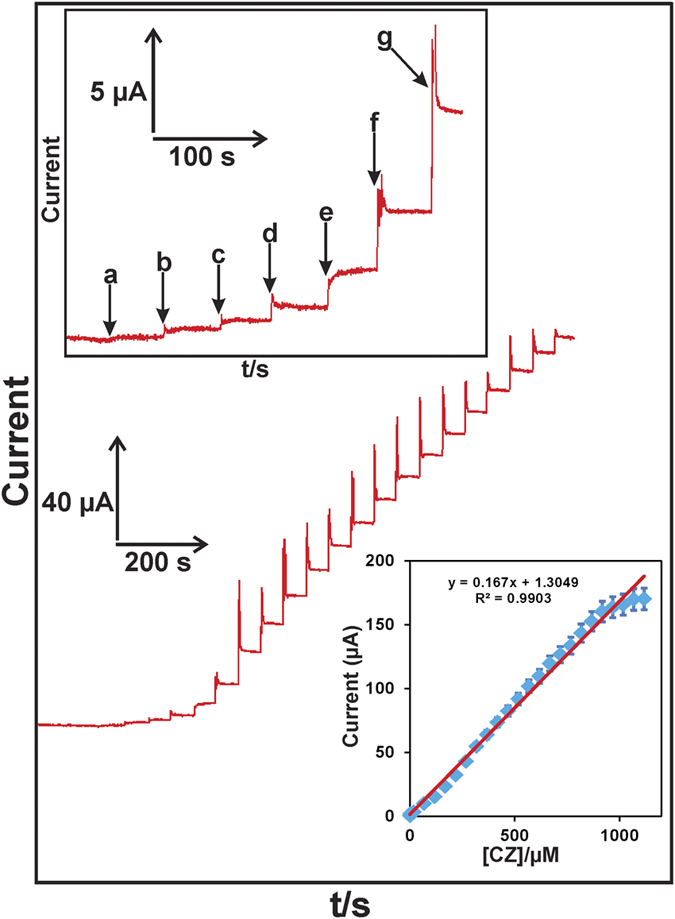
Amperometric *i-t* response of RGO@PDA composite modified RDE in the presence of different additions (0.003 to 1117.6 μM) of CPZ into constantly stirred N_2_ saturated PBS. Working potential = 0.8 V. Inset (upper) shows the enlarged amperometric response of RGO@PDA composite for the addition of 0.03 μM (a), 0.3 μM (b), 1 μM (c), 5 μM (d), 10 μM (e), 50 μM (f) and 100 μM (g) of CPZ into PBS. Inset (lower) shows the calibration plot for amperometric current response of vs. [CPZ].

To fabricate RGO@PDA composite modified electrode, 8 μL of GO@PDA composite solution was dropped on pre-cleaned GCE and dried in an air oven. Then, GO@PDA composite modified electrode was transferred into an electrochemical cell containing pH 5 and applied a constant potential of –1.4 V for 300 s. Finally, GO@PDA composite was transformed into RGO@PDA composite (Fig. 1), and the resulting composite electrode was dried at room temperature. For comparison, a modified electrode of RGO only was prepared by the reduction of GO modified electrode in pH 5 without PDA. All modified electrodes were stored in room temperature under inert conditions when not in use.

## Additional Information

**How to cite this article**: Palanisamy, S. *et al.* A Facile Electrochemical Preparation of Reduced Graphene Oxide@Polydopamine Composite: A Novel Electrochemical Sensing Platform for Amperometric Detection of Chlorpromazine. *Sci. Rep.* **6**, 33599; doi: 10.1038/srep33599 (2016).

## Supplementary Material

Supplementary Information
