# Supplementary material for: A Facile Electrochemical Preparation of Reduced Graphene Oxide@Polydopamine Composite: A Novel Electrochemical Sensing Platform for Amperometric Detection of Chlorpromazine
Source: Sci Rep. 2016 Sep 21;6:33599. doi: 10.1038/srep33599 (PMC5030524; doi:10.1038/srep33599)
Supplement: Supplementary Information [file srep33599-s1.doc]

**A Facile Electrochemical Preparation of Reduced Graphene Oxide@Polydopamine Composite: A Novel Electrochemical Sensing Platform for Amperometric Detection of Chlorpromazine**

Selvakumar Palanisamy^a^, Balamurugan Thirumalraj^a^, Shen-Ming Chen^a^, Yi-Ting Wang^a^, Vijayalakshmi Velusamy^b^ & Sayee Kannan Ramaraj^c^

^a^Electroanalysis and Bioelectrochemistry Lab, Department of Chemical Engineering and Biotechnology, National Taipei University of Technology, No. 1, Section 3, Chung-Hsiao East Road, Taipei 106, Taiwan, ROC.

^b^Division of Electrical and Electronic Engineering, School of Engineering, Manchester Metropolitan University, Manchester M1 5GD, United Kingdom.

^c^ PG & Research department of Chemistry, Thiagarajar College, Madurai-625009, India.

Correspondence and requests for materials should be addressed to S.M.C. (smchen78@ ms15.hinet.net)

**Supporting information**


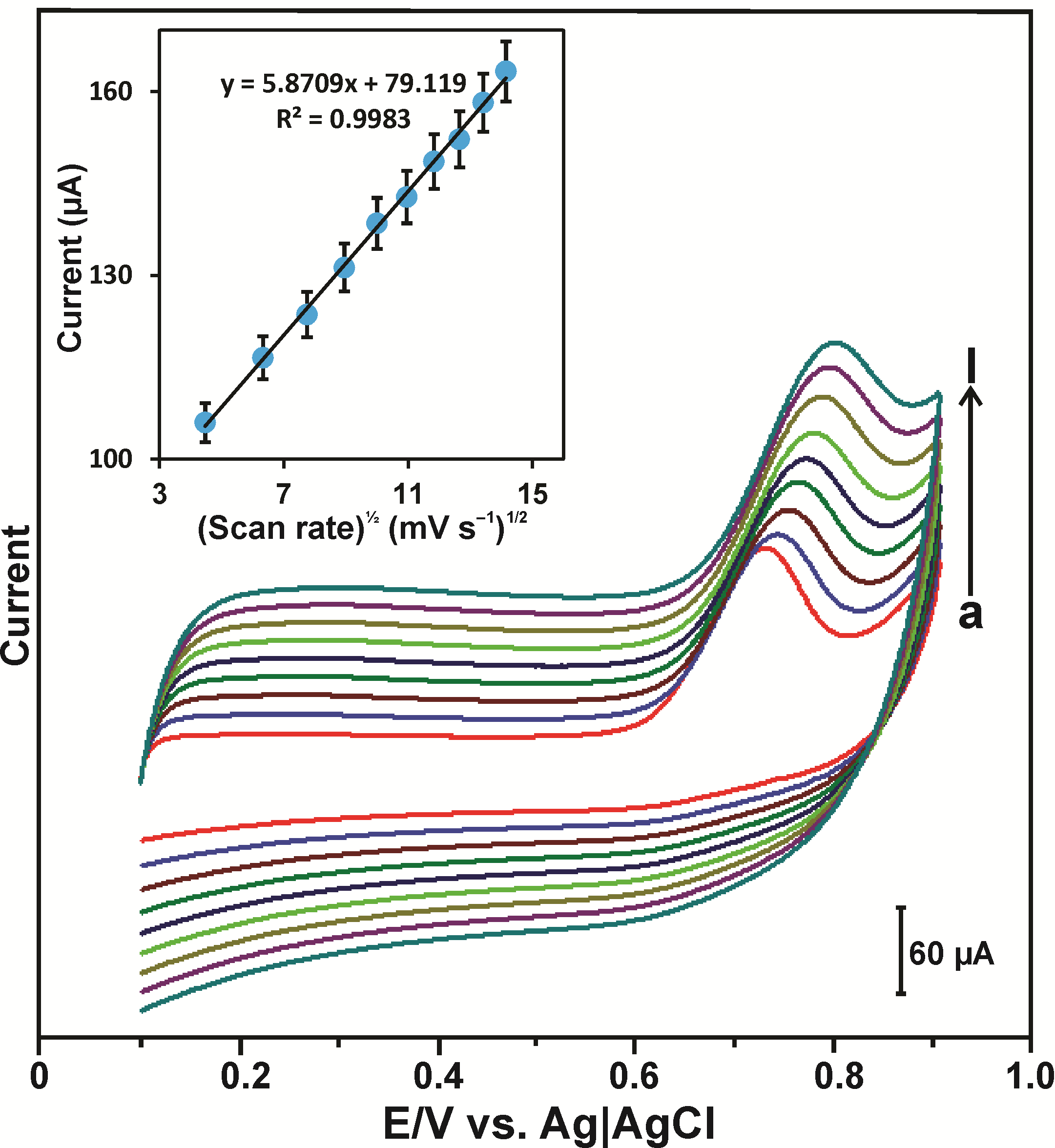
**Figure S1.** cyclic voltammograms of RGO@PDA composite modified electrode in N_2_ saturated PBS containing 2 mM of CPZ at different scan rates from 20–200 mV/s (a–i). Inset shows the linear dependence of oxidation peak current with the square root of scan rate.

**
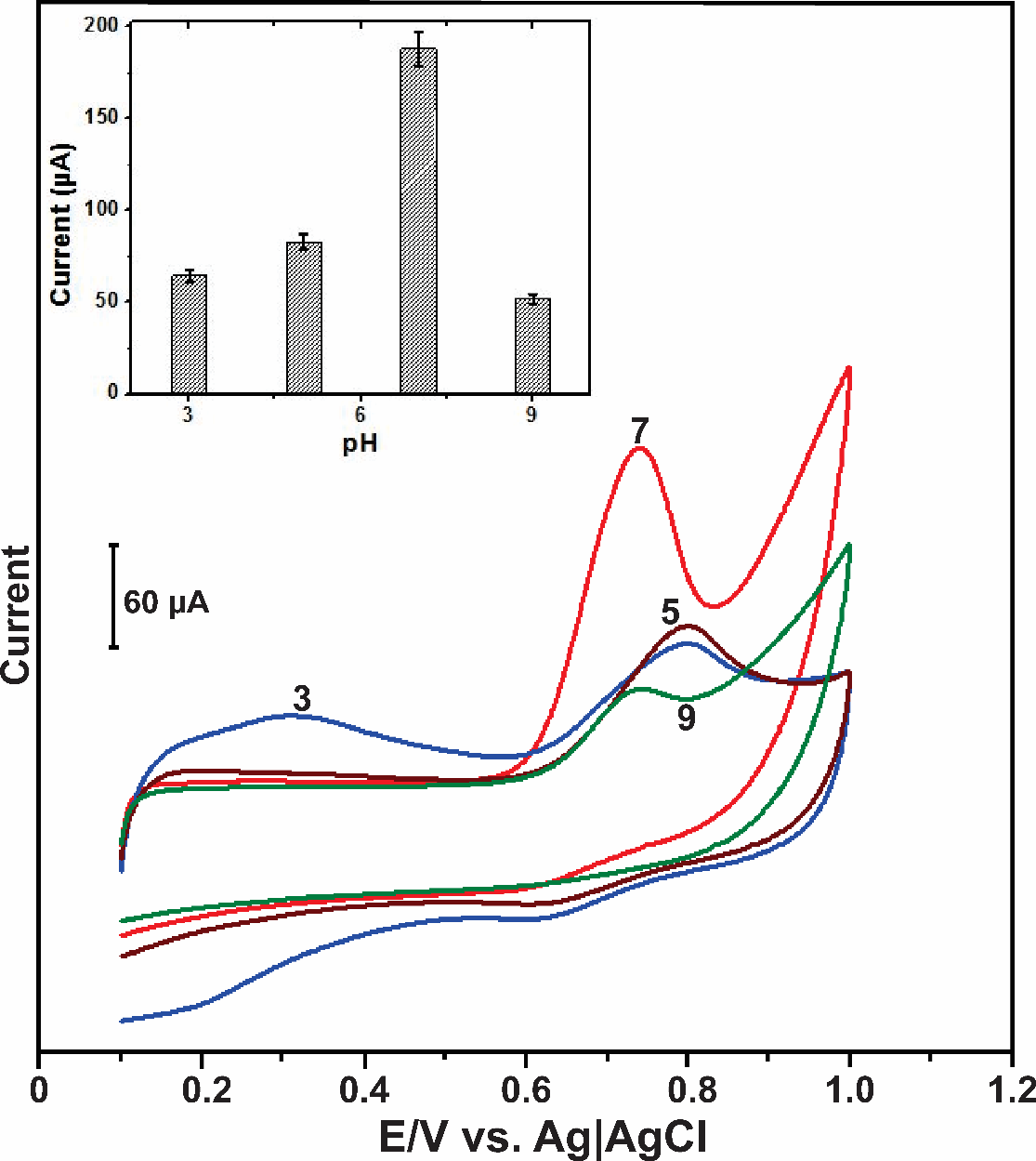
**

**Figure S2.** Cyclic voltammetry response of RGO@PDA composite modified electrode in N_2_ saturated different pH (pH 3, 5, 7 and 9) containing 2 mM CPZ at a scan rate of 50 mV/s. Inset shows the oxidation peak current response of CPZ in different pH. Error bars relative to the five measurements.


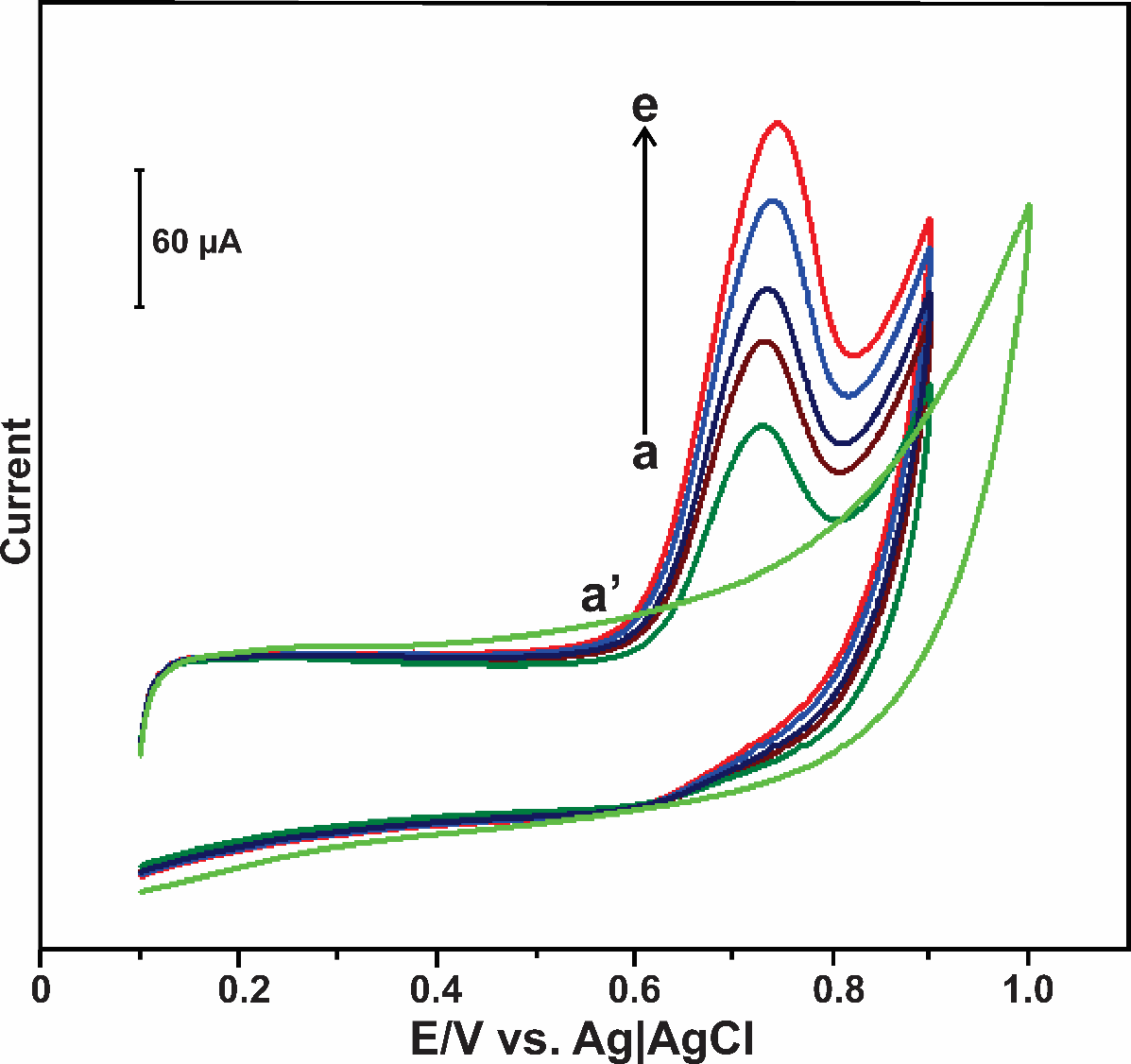


**Figure S3.** Cyclic voltammograms of RGO@PDA composite in the absence (a’) and presence of various concentrations of CPZ (a) 0.3, (b) 0.7, (c) 1.0, (d) 1.5 and (e) 2.0 mM in N_2_ saturated PBS.


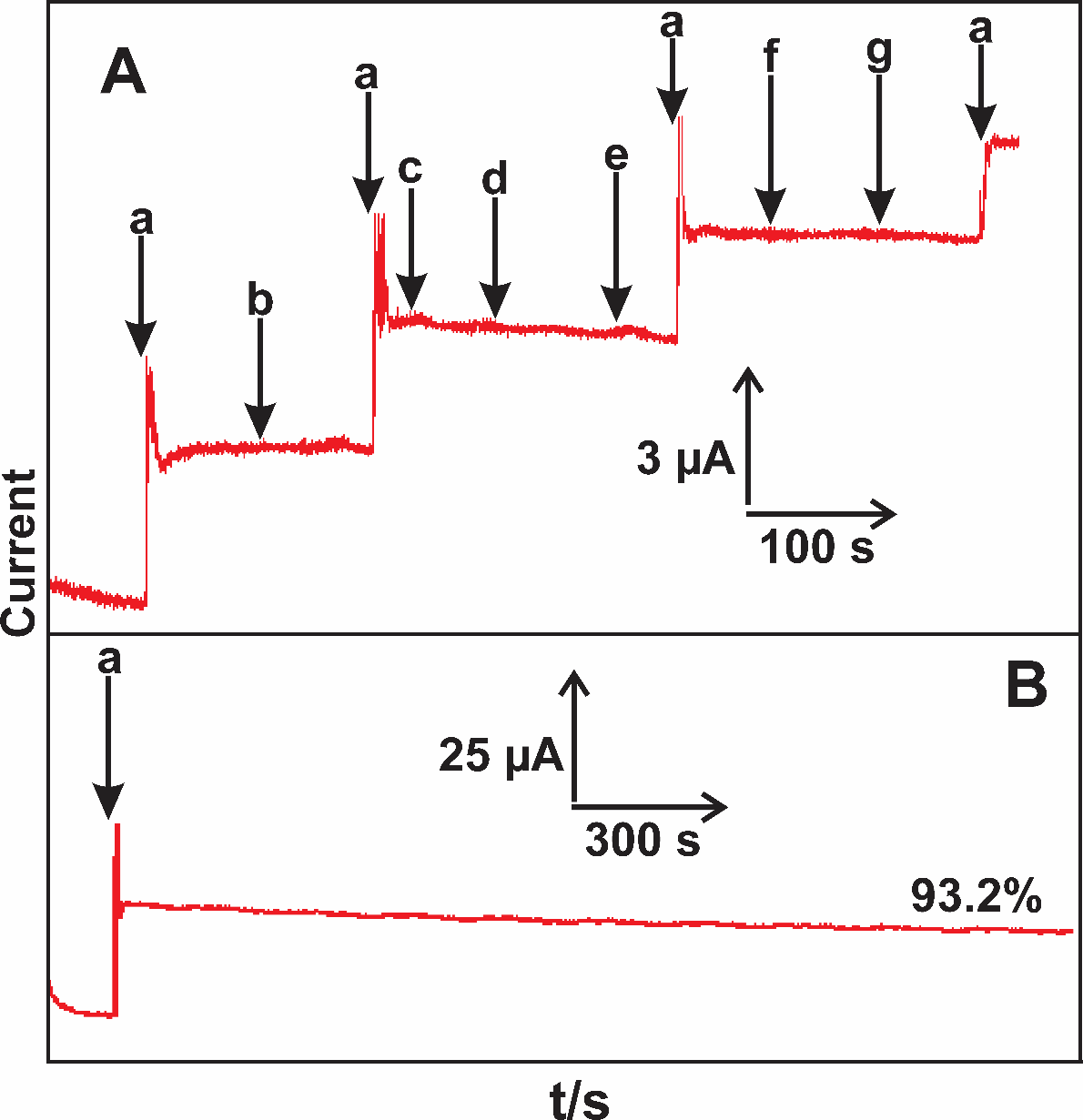


**Figure S4.** A) Amperometric *i-t* response obtained at RGO@PDA composite modified RDE for the addition of 1 µM of CPZ (a) and 100 µM of metronidazole (b), phenobarbital (c), chlorpheniramine maleate (d), pyridoxine (e), riboflavin (f) and ascorbic acid (g) into the constantly stirred N_2_ saturated PBS at the working potential of 0.8 V. B) Amperometric *i-t* response of RGO@PDA composite modified RDE for addition of 50 µM of CPZ (a) into the constantly stirred N_2_ saturated PBS and the background current response up to 2000 s; working potential = 0.8 V.

**Table S1** Determination of CPZ in WINSUMIN tablets using RGO@PDA composite electrode by amperometric *i-t* method. The RSD is related to the standard deviation of 3 measurements.

| **Sample** | **Added (µM)** | **Detected**  **(µM)** | **Recovery (%)** | **RSD** |
| --- | --- | --- | --- | --- |
| **WINSUMIN tablets (12.5 mg)** | - | 28.6 | - | - |
|  | 5.0 | 33.1 | 98.5 | 4.6 |
|  | 5.0 | 37.8 | 99.2 | 4.3 |
